# Automatic Bluefin Tuna Sizing with a Combined Acoustic and Optical Sensor

**DOI:** 10.3390/s20185294

**Published:** 2020-09-16

**Authors:** Pau Muñoz-Benavent, Vicente Puig-Pons, Gabriela Andreu-García, Víctor Espinosa, Vicente Atienza-Vanacloig, Isabel Pérez-Arjona

**Affiliations:** 1 Institute of Control Systems and Industrial Computing (AI2), Universitat Politècnica de València (UPV), 46022 València, Spain; gandreu@upv.es (G.A.-G.); vatienza@upv.es (V.A.-V.); 2 Institut d’Investigació per a la Gestió Integrada de Zones Costaneres (IGIC), Universitat Politècnica de València (UPV), 46022 València, Spain; vipuipon@upv.es (V.P.-P.); vespinos@upv.es (V.E.); iparjona@upv.es (I.P.-A.)

**Keywords:** underwater acoustics, underwater computer vision, fishery management, fish sizing, biomass estimation, automatic 3D measurements

## Abstract

A proposal is described for an underwater sensor combining an acoustic device with an optical one to automatically size juvenile bluefin tuna from a ventral perspective. Acoustic and optical information is acquired when the tuna are swimming freely and the fish cross our combined sensor’s field of view. Image processing techniques are used to identify and classify fish traces in acoustic data (echogram), while the video frames are processed by fitting a deformable model of the fishes’ ventral silhouette. Finally, the fish are sized combining the processed acoustic and optical data, once the correspondence between the two kinds of data is verified. The proposed system is able to automatically give accurate measurements of the tuna’s Snout-Fork Length (SFL) and width. In comparison with our previously validated automatic sizing procedure with stereoscopic vision, this proposal improves the samples per hour of computing time by 7.2 times in a tank with 77 juveniles of Atlantic bluefin tuna (*Thunnus thynnus*), without compromising the accuracy of the measurements. This work validates the procedure for combining acoustic and optical data for fish sizing and is the first step towards an embedded sensor, whose electronics and processing capabilities should be optimized to be autonomous in terms of the power supply and to enable real-time processing.

## 1. Introduction

The proposal behind our study is to design dual sensors to provide some fault tolerance, energy savings and low cost in continuous monitoring. There is currently great demand for sensors that allow us to explore the underwater world, and these types of sensors are necessary for the purposes of both scientific and commercial exploration. Knowledge of the physical and biological conditions of underwater ecosystems and populations, as well as their behavior in the face of overfishing of species and noise from propellers that generate electric energy, all makes it necessary to study them in order to provide knowledge and tools for the marine authorities to legislate on how to exploit this environment in the least aggressive way possible.

Optical sensors are very appropriate for developing accurate, low cost, and non-invasive methods to explore underwater ecosystems and in particular for estimating fish biomass, as demonstrated in recent years [1,2,3,4,5,6]. The International Commission for the Conservation of Atlantic Tunas (ICCAT) recommends in [7] the use of stereoscopic vision systems (two cameras in a side-by-side arrangement) to size live fish in order to control catches for tuna farming. Nevertheless, optical sensors and the corresponding image processing methods have to overcome demanding underwater conditions such as restricted visibility and temporal and spatial variations in lighting. These difficulties have restricted the development of fully automatic solutions and, as some authors have pointed out in [3,4,8,9,10], fully automatic methods for these tasks are still an open topic. The most widely used commercial stereoscopic systems for fish sizing, AQ1 AM100 (AQ1 Systems Pty Ltd., Hobart, Tasmania, Australia) [11] and AKVAsmart (formerly VICASS [12]) (AKVAGroup, Torvastad, Norway) require human intervention. This slows down the process, makes it laborious, introduces the variability of manual measuring and limits the number of samples that can be gathered to statistically represent the fish stock. In addition to cameras, other kinds of sensors have also been used for fish sizing, such as diode frames in [13,14], Light Detection and Ranging (LiDAR) systems in [15], acoustic cameras in [16,17], and acoustic sensors in [18]. These sensors have no limitations regarding visibility and lighting in underwater conditions, but further developments are required to attain individual fish sizing. To the best of the authors’ knowledge, proposals with optical sensors [4,6,8,9,19,20] have provided the best results so far when dealing with automatic fish sizing. However, videos contain such an amount of information that automatically processing the video data to recognize and size fish in images consumes a lot of computing time [21]. A common approach to overcome the limitations of optical sensors is to combine them with other kinds of sensors such as a camera with a laser [22,23], a camera with LiDAR [24], or a camera with an acoustic sensor [1,6]. In the particular application of fish biomass estimation, cameras have been previously combined with acoustic sensors in [25,26,27,28,29,30]. Electronic devices need power to function, and even more so if the scene needs to be illuminated by some source of light. Making these devices operate continuously entails equipping them with large batteries or replacing them frequently, which would greatly increase the cost of monitoring.

The purpose of this work is to combine the intrinsic benefits of acoustic systems in underwater environments with our recent developments [6,20,21] at the cutting edge of computer vision systems for fish sizing. In particular, this work exploits the benefits of the acoustic device to identify fish in the volume covered by the sensor and use that information to alleviate the computing time consumption of video processing algorithms and hence the power consumption. Acoustics could be used as a presence detector to activate video acquisition and possible sources of light only when an individual crosses the camera’s field of view. From the acoustic information, we can deduce the distance from the sensor to the individual, which together with the image of a single camera allows us to estimate the sizes of individuals. This paper presents an automatic sizing procedure based on computer vision techniques, able to accurately estimate a great number of samples using a sensor that combines acoustic and optical data. The system is able to automatically give accurate measurements of tuna Snout-Fork Length (SFL) and Width (W), while discarding samples that could lead to erroneous measurements by using a Convolutional Neural Network (CNN). In comparison with our previously validated automatic sizing procedure with stereoscopic vision [20,21], the proposal improves the samples-per-hour of computing time by 7.2 times in a 3500 m^3^ tank with 77 juveniles of Atlantic Bluefin Tuna (ABFT), without compromising the accuracy of the measurements. The results confirm the potential of the proposed automatic sizing method, taking a first step towards an Acoustic–Optical (AO) embedded sensor whose electronics and processing capabilities should be optimized to have an autonomous power supply and to enable real-time processing. Although the system is evaluated in an aquaculture environment for a single species, this is only the first step towards generalizing its functionality for a wild underwater environment and other species.

## 2. Materials and Methods

**Figure 1:**
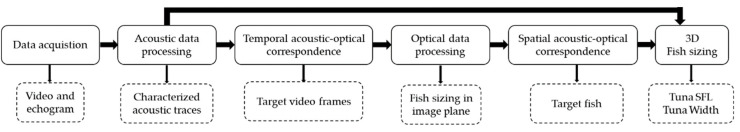
Top row: Sequence of processes performed automatically in our proposal; bottom row: The results of each step.

The algorithms involved in the automatic process of fish sizing are described and summarized in Figure 1.

**Figure 2:**
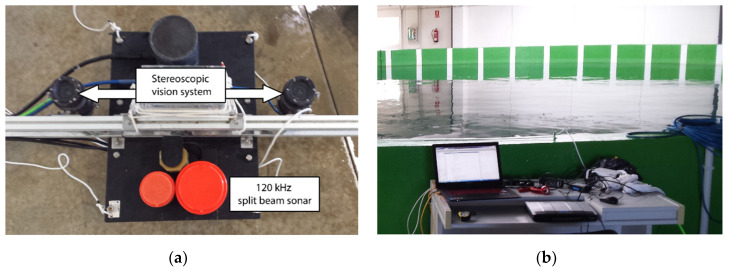
(**a**) Sensor platform equipped with a stereoscopic camera and a 120 kHz split beam sonar, among other sensors, (**b**) Logging computer placed close to the tanks to register the recordings.

The equipment used to acquire acoustic and visual data is shown in Figure 2 and explained below in Section 2.1. The algorithms for acoustic and optical data processing are detailed in Section 2.2 and Section 2.3, respectively, whereas the spatial and temporal correspondence between acoustic and optical data, as well as the combination of them to obtain 3D fish measurements, is explained in Section 2.4.

### 2.1. Data Acquisition

The recordings were taken at the Infrastructure for Atlantic Bluefin Tuna Aquaculture (ICAR), belonging to the IEO (Spanish Oceanographic Institute). The ICAR is a unique scientific and technical infrastructure (ICTS) devoted to studying the complete aquaculture of ABFT. A sensor platform (Figure 2a) was placed in one of the tanks—which measure 20 m in diameter, 10 m in depth, and 3500 m^3^ in volume—containing 77 ABFT juveniles in sea water. The platform was equipped with a stereoscopic camera and a 120 kHz split beam sonar, among other sensors. It was positioned lying on the bottom of the tank and looking towards the surface in order to have a ventral perspective of the fish. Thirty-three hours were recorded from 24 May to 26 May and fifty hours from 11 September to 16 September.

The 120 kHz split-beam transducer (Simrad ES120-7C, Kongsberg Maritime AS, Horten, Norway) operated by a Simrad EK80 (Kongsberg Maritime AS, Horten, Norway) echosounder was set up with a transmitting power of 100 W, pulse length of 64 µs and 20 pings per second. The nominal acoustic beam angle was 7°. The on-axis and off-axis calibration was carried out using the standard calibration method, with a 23 mm diameter copper sphere [31].

Video recordings were taken with a customized stereo camera comprised of two Gigabit Ethernet cameras, with a 1720 × 1080 pixel resolution and framerate of 35 fps. The cameras were mounted in an underwater housing, with a baseline of 85 cm and inward convergence of 5°. Camera synchronization was achieved using the IEEE 1588 Precision Time Protocol (PTP) [32]. The system is rated for a depth of 40 m and has an umbilical cable that supplies power over ethernet to the cameras and transfers images to a logging computer (Figure 2b), which encodes left and right videos using GPU encoding. The stereoscopic system was previously calibrated using a checkerboard pattern and the MATLAB^®^ Stereo Calibration Application based on [33] and [34].

To analyze the feasibility of the proposed AO sensor, the data from the acoustic transducer and one of the cameras of the stereoscopic pair was to be merged and processed to estimate the fish size.

### 2.2. Acoustic Data Processing for Trace Identification and Characterization

**Figure 3:**
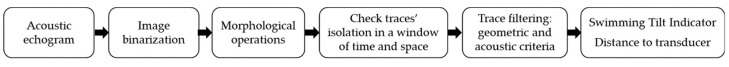
Sequence of acoustic echogram processing algorithms involved in the process of trace identification and characterization.

**Figure 4:**
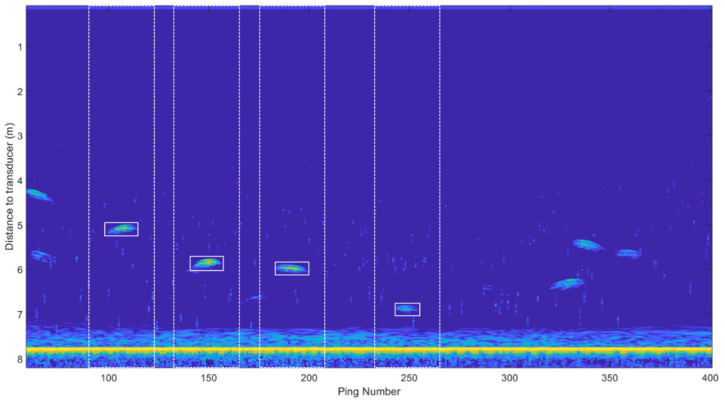
Identification of acoustic traces in the echogram within windows of time and space.

**Figure 5:**
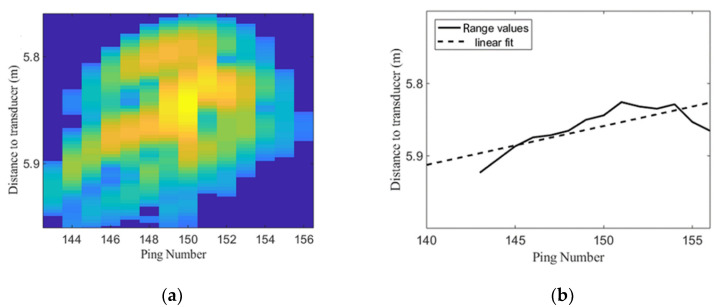

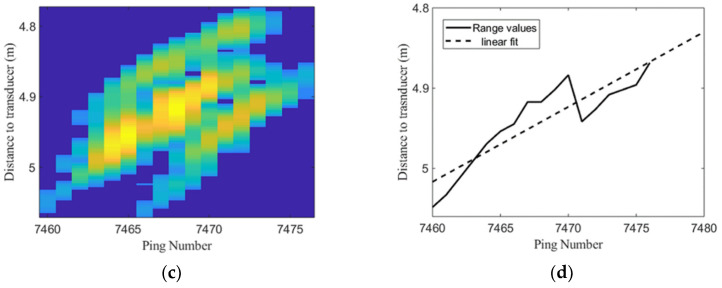
(**a**) Trace of fish with swimming tilt indicator (STI) = 0.07, (**b**) Range values and linear fit from trace in (**a**), (**c**) Trace of fish with STI = 0.53, (**d**) Range values and linear fit from trace in (**c**).

The automatic procedure for acoustic data processing is based on applying image processing techniques to acoustic echograms and is summarized in Figure 3. Due to the acquisition settings, different complex shapes of traces were recorded (see Figure 4 and Figure 5 for examples of traces). Those shapes depend on multiple factors, such as the insonification angle, distance to the transducer and tuna swimming tilt angle, among others.

In the first step, the echogram was transformed into a binary image using the threshold level defined by Otsu’s method [35]. In the second, a sequence of morphological operations was applied: thickening to provide more compact traces, opening to remove protrusions (noise), breaking weak connections, and closing to smooth out contours and fill small holes. Traces that were not isolated in a window of time and space in the echogram were discarded, since they may lead to ambiguity in the acoustic–optical correspondence, as explained in Section 2.4. Then, the traces were geometrically characterized and filtered using the two criteria of area and solidity. Finally, acoustic properties were analyzed to differentiate good quality traces based on the following parameters: maximum and minimum TS (target strength) value, distance to the transducer and number of pings. The result of the acoustic data processing is a collection of traces characterized by their shape and the distance to the transducer. Figure 4 shows the identification of acoustic traces in the echogram within windows of time and space. Note that traces that were not isolated in those windows, for example, around ping number 350, were discarded. The windows of time and space in the echogram have a rectangular form: the width corresponds to the time dimension and the height to the space dimension (distance to the transducer). The width is established after a preliminary analysis, which consists of computing the average duration of a subgroup of traces, whereas the height covers the entire water column. The windows of time and space are thus defined by rectangles whose sides are 1000 ms (traces average duration plus margins) and 8 m (water column). The values were fixed beforehand, and they do not interfere with a possible real-time application.

Additional information can be deduced from acoustic traces, for example, a Swimming Tilt Indicator (STI). The maximum backscattering value of each ping of the trace is calculated and the distance or range from each maximum to the transducer is obtained (Figure 5b). A linear fit is applied to the range values and the slope of the line is used as the STI. Figure 5a,b shows an example of a trace with a low STI, whereas Figure 5c,d shows an example of a trace with high STI. The indicator will be used in Section 2.4 to transform image plane measurements to 3D sizes and in Section 3.1 to discriminate fish depending on the swimming tilt angle.

### 2.3. Optical Data Processing for Fish Sizing in Images

**Figure 6:**
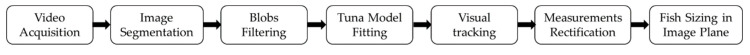
Sequence of video processing algorithms involved in the process of fish sizing.

**Figure 7:**
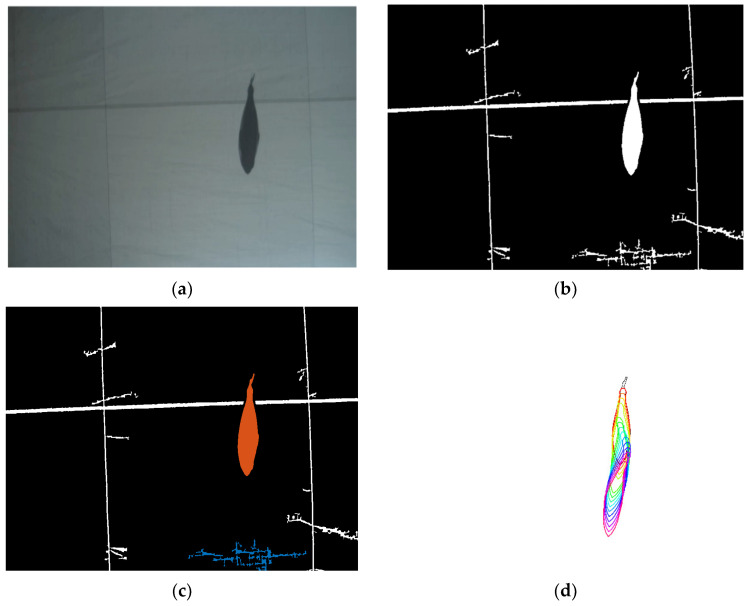
Optical data processing. (**a**) Original image, (**b**) segmented image using local thresholding and morphological operations, (**c**) blob labelling and filtering, and (**d**) tuna model fitting and visual tracking.

The computer vision algorithms involved in optical data processing are summarized in Figure 6 and illustrated in Figure 7. The optical data is composed of frames of the videos acquired in ICAR tanks in real conditions. The image segmentation is implemented using local thresholding [36], a region-based technique for extracting compact regions (blobs) in each video frame, and morphological operations. The segmented blobs are geometrically characterized and filtered using shape (aspect ratio), pixel density, and dimensional filters. An edge detection algorithm is then applied, and a minimization algorithm is used to fit a deformable tuna model, from which fish measurements are deduced. The deformable tuna model was defined in [21] as a vector of eight parameters M = $\left[s_{x},s_{y},l,\alpha ,\theta ,w,l_{p},s_{p}\right]$, where: $s_{x}$ and $s_{y}$ give the image location of the snout tip; l is the length of the spine; *α* denotes the angle of the fish’s head in relation to the horizontal axis, and *θ* is the global bending angle of the spine; w is the widths vector; and $\left[l_{p},s_{p}\right]$ are the length and slope of the segment representing the back part of the caudal peduncle. A Fitting Error Index (FEI), based on the quadratic distance between the model points and target edge points is used to confirm good model fittings. See [10,20,21] for further details on the computer vision algorithms.

A new innovation introduced with respect to our previous works is the visual tracking, which allows us to obtain reliable size measurements based on the repetition of several measurements of the same fish. This visual tracking is based on the fact that once fish are appropriately identified in the video frames and tuna models are fitted to their silhouettes, measurements are considered to belong to the same fish when silhouette models overlap in neighboring video frames and have similar lengths and swimming directions. Fish measurements are computed using trimmed means, i.e., means excluding outliers, and rectified using the calibrated intrinsic camera parameters. An example of our visual tracking can be seen in Figure 7d, where one fish is identified and measured 24 times (in 24 almost consecutive frames). Note that the GPU encoding mentioned in Section 2.1 allows us to have a high framerate (35 fps) and to process several images of the same fish.

The explanation for mapping image plane (2D) sizes to the 3D world by combining acoustic and optical information is given in the following Section 2.4. In our previous works, when stereoscopic cameras were used, results from left and right videos were merged to size the fish using stereoscopic correspondence and epipolar geometry. However, with the proposed sensor composed of only one camera, the video processing algorithms culminate with the fish sizing in the image plane.

As for the application of our algorithms (which involves automatic video processing) to these experiments, we would like to mention that the indoor facilities brought us obvious advantages in terms of comfort, but introduced other difficulties that were not present in our previous works in outdoor facilities. One of the most relevant changes concerned image segmentation: the fish were difficult to segment from the background using the previously adopted local thresholding since the background was not uniform. Therefore, a white back panel was installed to guarantee a uniform background suitable for automatic measurements, as can be seen in Figure 7a. On our earlier outdoor experiments [6,10,20,21], the ventral perspective was chosen due to its advantages: first, the sunlight acted like a backlight system so objects are always darker than water; and second, body bending can be clearly appreciated and dealt with. Although the first advantage is not met in indoor facilities, the ventral perspective is still preferable to benefit from previous knowledge regarding image segmentation and to apply our previously developed deformable tuna model. However, as will be noticed in Section 4 as further work, we plan to work on other perspectives in addition to the ventral one. For the dorsal perspective (cameras looking downwards), the deformable model can be straightforwardly applied, since the ventral and dorsal silhouettes are identical, but we should ensure that the fish is clearly distinguishable from the ground to use the local thresholding segmentation. In the current case of indoor facilities, this means that the bottom of the tank should be cleaned, or a back panel should be placed, similarly to the one placed for the ventral perspective. For the lateral perspective, the deformable tuna model should be adapted, or another strategy should be applied.

### 2.4. Combination of Acoustic and Optical Processing for 3D Fish Sizing

In order to combine information from both the acoustic and optical devices, spatial and temporal correspondence must be ensured, i.e., it must be ensured that traces in the acoustic echogram and the fish in the images correspond to same fish.

As regards the temporal correspondence, both acoustic and optical data are acquired with the same logging computer, so timestamps are related to the same computer’s clock. When fish traces are identified and characterized as explained in Section 2.2, bounded windows of time of 1000 ms are defined around the instant of the centers of the traces, from 500 ms before the centers to 500 ms after them. The corresponding video frames are then located in the recordings and the optical processing algorithms are applied to size the fish in the image plane. The acoustic data acts as a kind of trigger for video processing or a motion detector in the camera’s field of view.

**Figure 8:**
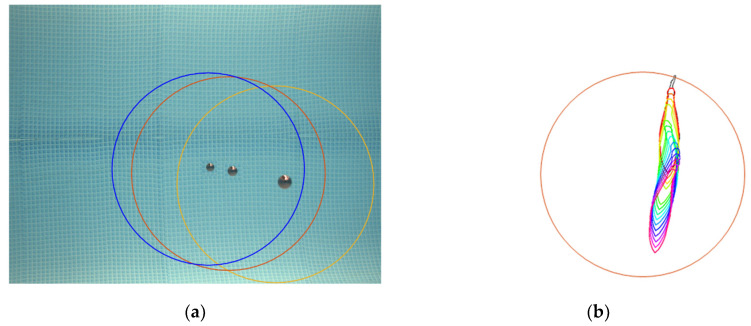
Spatial and temporal Acoustic–Optical (AO) correspondence. (**a**) AO coarse extrinsic calibration: verification of the projection of the acoustic beam onto the image for different ranges, and (**b**) fish measured in in the insonified area and in the window of time around the instant of the acoustic trace.

The spatial correspondence is met when a fish intersects the projection of the acoustic beam in the image. It does not need to be fully contained in the projection, because only traces isolated in a window of time and space in the echogram are considered, as explained in Section 2.2. To deduce the relative position and orientation between acoustic and optical devices and deal with experimental assembly inaccuracies, a coarse extrinsic calibration between camera and transducer is carried out. The purpose of the calibration is to locate the projection of the acoustic beam onto the image for different distances in order to find the equivalent insonified area in the image. The procedure is as follows: The sensor is placed in a fixed position and a calibration sphere hangs from a string attached to a 3D high-precision movable axis. The sphere is aligned with the echo sounder beam at different distances, while images from the camera are captured. Knowing the position of the sphere, the distance to the sensor given by the echo sounder and the aperture of the acoustic beam (7°), the latter can be projected onto the image. The projection of the acoustic beam onto the image can be narrowed down to restrict the spatial correspondence at the expense of decreasing the number of correspondences. Figure 8a illustrates the projected acoustic beam onto the image for different distances and Figure 8b represents the fish identification for sizing when the temporal and spatial AO correspondence are met.

When the temporal and spatial correspondence between acoustic and optical data has been ensured, the image plane measurements can be transformed to 3D measurements using the acoustic range. Firstly, sizes in pixels are rectified using the intrinsic camera parameters, so they can then be transferred into the 3D world using the well-known pinhole camera model and similar triangles:

(1) $$Y=\frac{y}{f}Z$$

where Y is the size in meters, y is the size in pixels, f the camera’s focal length and Z the rangein meters.

Thanks to the tracking algorithm explained in Section 2.3, we dispose of several measurements of the same fish in the image plane. Thus, to have more reliable fish measurements, the following trimmed mean is proposed:

(2) $$Y=\frac{\sum_{i=p+1}^{n-p}\frac{y_{i}}{f}Z_{i}}{n-2p}$$

where n is the number of measurements of the same fish, p is a fixed number that represents the amount of values considered outliers for the trimmed mean, $y_{i}$ is the size in pixels of each measurement, and $Z_{i}$ their corresponding range. For each measurement, the image timestamp is related to the trace ping number, which is used together with the STI presented in Section 2.2 to find $Z_{i}$. For each fish, 3D sizes are computed from the sizes in pixels $y_{i}$, their corresponding $Z_{i}$ and the trimmed mean.

The fish are sized in two dimensions—SFL and maximum width—which are deduced from the fitted model. The tuna’s maximum width corresponds to the first element in the width vector (**w**), whereas the tuna’s length in the image plane corresponds to the length of the spine (*l*). They are transferred into the 3D space as Width (W) and Model Length (ML) using Equation (2). Note that the caudal peduncle is not included in the tuna model due to its great variability, so SFL needs to be calculated from the Model Length (ML) by using the relation SFL = 1.0312 ML + 0.065641, deduced from experimental samples in [20].

### 2.5. Discarding Measurements with High Swimming Tilt Angle

In an ideal case, where fish swim perpendicular to the camera axis, the silhouette and sizes of fish would be projected onto the image with no distortion in perspective. However, the distortion increases with the swimming tilt angle, which can lead to inaccurate estimations when using only one camera. Fish swimming tilt angle (θ) can be easily computed using a stereoscopic vision system, once the fish is detected in the two stereoscopic frames. When the points of the tuna’s snout and fork are identified and mapped onto the 3D world, the angle can be deduced using trigonometry. However, this cannot be inferred from monoscopic video, so instead we propose to use the echogram traces to deduce it, so the measurements can be restricted to cases with a low perspective distortion, i.e., to cases where fish swim with a low tilt angle. Two different approaches are tested to identify tilted traces (|θ| > 10°) in order to dismiss them: the first one, constraining the STI explained in Section 2.2 to a delimited interval, and the second one, applying deep learning techniques, in particular training and using a Convolutional Neural Network (CNN) by applying transfer learning with an AlexNet network.

Deep learning techniques have been widely used for computer vision applications in the last years [37,38]. CNNs constitute a class of deep, feed-forward Artificial Neural Networks (ANN) [39]. A CNN architecture is structured basically in convolution and pooling layers. The convolutional layers act as feature extractors from the input images whose dimensionality is then reduced by the pooling layers. The convolutional layers encode may be understood as banks of filters that transform an input image into another, highlighting specific patterns. The output of the CNN models is structured in fully connected layers, which act as classifiers exploiting the high-level features learned to classify input images in predefined classes or to make numerical predictions. These fully connected layers take a vector as input and produce another vector as output. A disadvantage of deep learning is the generally longer training time; however, they are super-fast on testing time [40]. Transfer learning [41,42] is a very popular technique in deep learning, which takes advantage of previously tested and trained networks with millions of data to adapt them to new tasks with much faster training. This makes it possible to considerably shorten the development time and to obtain good results with a limited number of images. AlexNet [43] is a popular architecture used by researcher to start building their models, but a fine-tuning stage to adapt to specific tasks and labelled datasets of the new training data is crucial and necessary [38].

## 3. Results

For a quantitative evaluation of the proposal, the results obtained with the AO sensor were compared with the results obtained with the stereoscopic system from the points of view of accuracy, computing time, number of measurements and stock characterization with frequency histograms. The analysis of these values will demonstrate the potential advantages of using our system, thus validating the proposal. For this reason, the acquired stereoscopic videos were processed with the automatic procedure validated in our previous works [20,21] to generate valid ground truths, whereas AO data was processed with the proposed method to generate datasets. In Section 3.1, fish length measurements are analyzed to compare the accuracy of both systems; in Section 3.2 the computing time and quantity of measurements are compared; and in Section 3.3 frequency histograms to represent fish stock are examined.

### 3.1. Accuracy Analysis

The first analysis was carried out with 6031 samples (3439 samples gathered in May and 2592 samples in September) that were measured both with the stereoscopic system and the AO sensor to examine discrepancies in accuracy between the two systems. The relative error between measurements was defined and calculated for each sample as stated in Equation (3), where AO and S are the acoustic–optical and stereoscopic sizes (SFL and W), respectively.

(3) $$e_{r}\left(\%\right)=\frac{\mathrm{AO}-S}{S}\cdot 100$$

**Figure 9:**
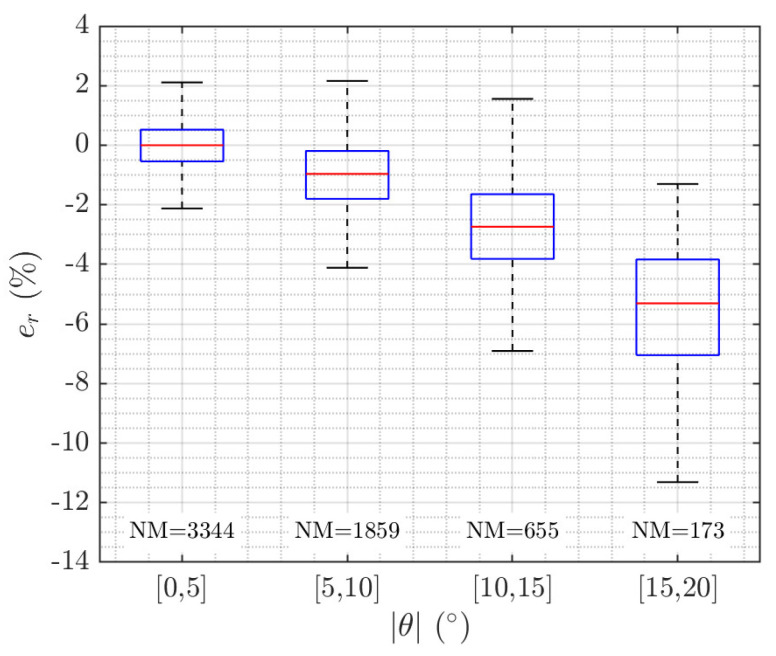
Relative error (e_r_) between acoustic–optical (AO) and stereoscopic measurements depending on the swimming tilt angle (θ). NM: number of measurements.

Figure 9 shows relative errors in length classified depending on the absolute value of the swimming tilt angle using a box plot. For each box, the central rectangle represents the interquartile range or IQR, which accounts for 50% of the samples, from the 25th to 75th percentiles. A segment inside the rectangle shows the median error and whiskers above and below the box comprise 90% of the samples, from the 5th to 95th percentiles. As can be observed, the error greatly increases with the swimming tilt angle, but it is low when the measurements are constrained to a low angle range. When the measurements are constrained to $\left|\theta \right|\in {\left[0,5\right]}^{{}^{\circ}}$, then 50% of the samples have less than ±0.5% error and 90% of the samples have less than ±2% error, whereas when they are constrained to $\left|\theta \right|\in {\left[5,10\right]}^{{}^{\circ}}$, 50% of the samples lie in the [−2,0]% error margin and 90% of samples in the [−4,2]% margin. Therefore, from this experiment it can be concluded that the AO measurements must be constrained to cases with $\left|\theta \right|\in {\left[0,10\right]}^{{}^{\circ}}$.

**Figure 10:**
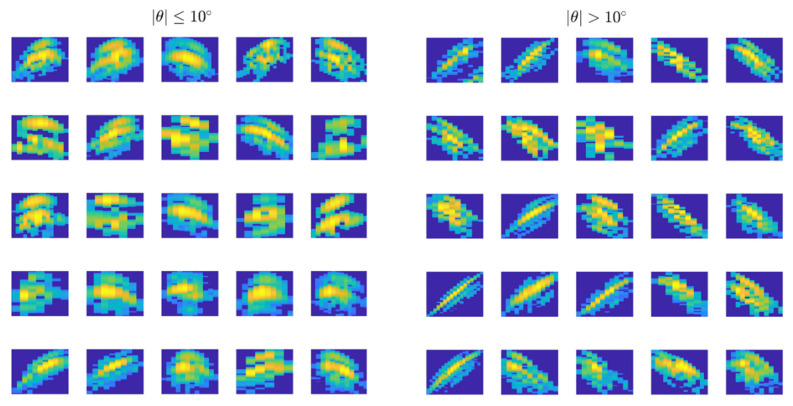
Sample of acoustic traces used in CNN training to discriminate tilted samples |θ| > 10° in the Acoustic–Optical (AO) measurements.

The two different approaches presented in Section 2.4 are tested to identify traces with |θ| > 10° in order to dismiss them: the first one consists in constraining the STI to a delimited interval, and the second one, in training and using a CNN. Measurements are grouped and labelled as tilted (|θ| > 10°) or non-tilted (|θ| ≤ 10°) samples, according to the swimming tilt angle computed with the stereoscopic system, and the classification rate between predicted and true labels is analyzed. The resulting dataset for fish sizing is made up of true positives from non-tilted samples and false positives from tilted samples. It is worth mentioning that the classification rate is particularly important when dealing with tilted samples, since a poor classification would lead to more false positives in the dataset used for fish sizing, which in turn can lead to wrong estimations. On the other hand, an over estimation of non-tilted samples decreases the number of samples in the dataset, but since the system supplies a high number of measurements the dataset is still statistically representative of the fish stock. As shown in Table 1, a proper classification (true positives) of between 56% and 71% is accomplished using STI, depending on the subgroup and the constraint interval. When STI∈[−0.4,0.4], there are 37% of the tilted samples classified as false positives, but the number of non-tilted samples falls from 5203 to 3486. When STI∈[−0.3,0.3], the percentage of false positives in tilted samples drops to 29%, but the number of non-tilted samples also drops to 2914. In situations where large datasets can be collected, and thus a high number of measurements can be obtained, STI∈[−0.3,0.3] would be preferable to limit the number of false positives. However, tilted samples are better discerned with the CNN, as at least 82% of targets are properly classified using only 50 traces of each subgroup for network training. When 200 images of each subgroup are used for training, the success rate increases to 85–86%, which suggests that if a greater number of samples were available it would increase further. Only 14–16.5% of the tilted samples are classified as false positives. To corroborate the prediction results from CNNs, the training was repeated 20 times with randomly selected images, and the average gives the prediction values. Note that the images used for network training are pulled out from the dataset and not classified in the prediction/true label columns. When the CNN is applied separately for each month, similar results in true positive classification are obtained, as shown in Table 2. We plan to extend the training set with new recordings from other months and sizes to make the detection more robust and ensure that CNN scale well with different sizes. Figure 10 shows 25 images of each subgroup to visualize the kind of acoustic images used to train the CNN. The results show that both STI and CNN approaches can be used to discard tilted samples, though the lower percentage of false positives added to the dataset indicates that it is advisable to use CNNs.

### 3.2. Computational Cost and Number of Measurements

Many aspects must be taken into account when the objective is to produce tools that automatically process information acquired in real conditions or natural environments. However, once the tool is achieved, two particularly important aspects must be assessed: the computational cost and the amount of information that can be extracted, in this case the number of individual fish that our sensor can identify and measure. In this section, AO and stereoscopic measurements are compared in terms of the number of measurements and computational cost. Although the stereoscopic algorithms and the hardware implementation can be optimized, the improvement shown in Table 3 that the AO sensor brings to the automatic measurements in terms of computing time, and hence in power consumption, is substantial. However, the number of measurements per recording is lower with the AO system. The experiments were run using MATLAB^®^ on a PC with an Intel Core i7-4790 processor at a clock frequency of 3.6 GHz.

On the one hand, the computational cost is lower because images from acoustic echograms contain less information than videos. Analyzing the acoustic echogram and the frames from one camera in the window of time given by the temporal correspondence is faster than analyzing a full stereoscopic video, although the algorithms applied to each video frame are almost the same, as explained in Section 2.3. The computational cost associated to the use of a trained CNN to discard tilted samples is very low compared to the total computational cost: classifying the 6031 samples lasts 106 s (17.6 milliseconds per sample). As mentioned in [40], a disadvantage of deep learning is the generally long training time; however, they are super-fast on testing time. In our case, the training of the network is performed offline. On the other hand, the number of samples is reduced because only isolated fish are analyzed. For example, when two fish swim very close to each other, there is no possibility to infer in the image which of them corresponds to each trace in the echogram. To guarantee temporal and spatial correspondence, traces that can lead to misunderstandings are discarded. Moreover, titled samples are discarded using CNN approach explained in 3.1 and the aperture of the acoustic beam (7°) is narrower than the cameras’ field of view (40°). However, the number of samples per hour of computing time is 7.2 times higher at 60.6 samples/hour compared to 9.3 samples/hour.

### 3.3. Stock Biomass Estimation

**Figure 11:**
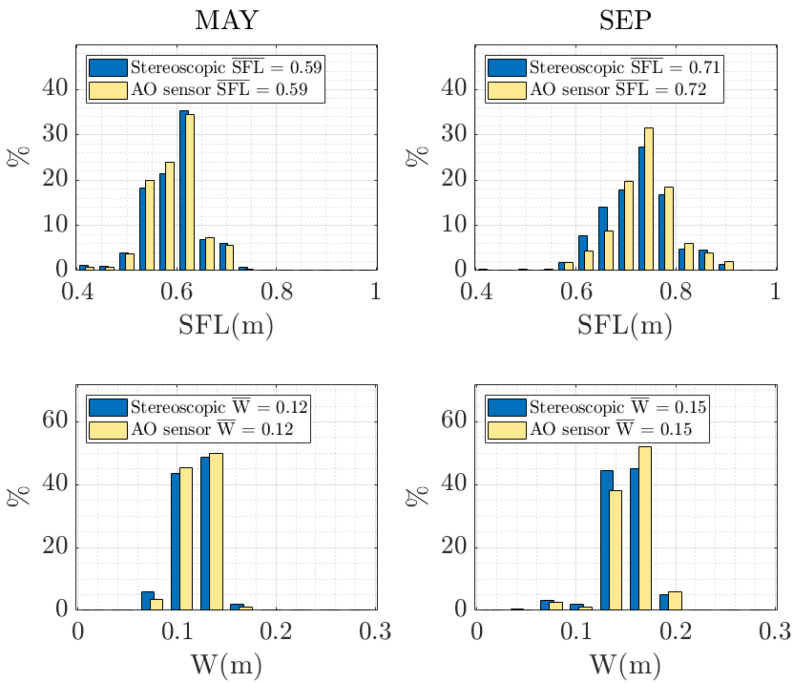
Snout fork length (SFL) and width (W) frequency histograms with the measurements from the acoustic–optical (AO) sensor and the stereoscopic system.

In this section, a statistical analysis is done to compare AO and stereoscopic measurements, and hence conclude about the validity of the systems to estimate stock biomass. The main statistical indicators, i.e., mean, standard deviation and variance, are presented in Table 4, whereas SFL and W frequency histograms are used to build up a statistical representation of fish stock (Figure 11). It can be seen that the mean values obtained with the stereoscopic system and the AO sensor differ by only 0–1 cm in length and 0 cm in width, and that the fish stock increased its size by 12–13 cm in length and 3 cm in width. Note that good results are obtained in samplings made at different times of year and different fish growth stages, which validates our system as a proper tool to monitor fish growth and study the possibility of defining growth models for different species—one of the most significant matters for farmers, biologists, and researchers [44]. Small discrepancies between AO and stereoscopic histograms occur because the same 77 fish are randomly swimming through the sensors’ field of view. However, when the number of measurements reach thousands of samples, the statistical indicators tend to stabilize. To overcome this issue, we are currently working on a tagging system able to identify each individual.

## 4. Conclusions

The dual sensor proposal has achieved good behavior in mapping the detected fishes’ 2D size onto the 3D world and has reduced the computational cost compared to the stereoscopic system. However, correct functioning of this duality implies greater complexity, so that our developments had to tackle and overcome various difficulties. One of them was to determine when the acoustic and optical information corresponds to the same individual, which is crucial in getting a correct sizing. Angular positions calculated from the split-beam system are not fully reliable due to fish size (lengths of between 0.50 and 1 m) and close range (2 to 6 m). The targets occupy more of one quadrant in the same instant, they are not punctual sources of backscattering, and the phase calculator can present error on its estimation. Hence, the acoustical analysis does not take into account this information, and the applied method is applicable to the output of any single-beam echosounder. Another important aspect for sizing is to obtain measurements when the visualization of the fish in the image is not affected by a distortion in perspective, i.e., when the fish are swimming with a low tilt angle. To discard fish swimming with high tilt angles, a CNN was trained to automate decision-making. The results and measurements obtained with this proposal are compared to those obtained with a previous design in which stereoscopic videos were used, as shown in Figure 9 and Figure 11 and Table 3. The use of CNN to identify the swimming angle versus the estimation based on acoustic STI has led to an increase in the total number of successes, reducing false positives (see Table 1), which avoids errors in size measurements. Our dataset has a larger number of fish samples with a swimming tilt angle |θ| ≤ 10° compared to those with |θ| > 10°; however, an equitable training set (50 + 50, 200 + 200) has been used in the experiment carried out. Furthermore, we have designed a tracking algorithm based on temporal and spatial information, providing reliable and more accurate size measurements based on the repetition of several measurements of the same fish. It is important to note that our sensor and automatic processing of acoustic and optical information provides not only length measurements (SFL) of tunas, but also width measurements (W), which requires an additional computational time cost.

Fish length information is an important indicator of the health of wild fish stocks and for predicting biomass using length–weight relationships [9,45], although recent studies have attempted to show that biomass can be estimated more accurately if fish measurements in dimensions other than length (such as width and height) are available [19,44,46]. The total biomass of a fish stock is commonly determined by obtaining the mean length of a statistically representative number of fish [8,47]. The most widely used commercial systems for biomass estimation are AQ1 AM100 [11] and AKVA smart (formerly VICASS [12]) but both have a significant limitation: they require human interaction. In both systems, human operators must inspect the videos, select the samples, and manually mark them with a mouse click. This process is slow and laborious, introduces the variability of manual measuring into the biomass estimation, and limits the number of samples.

Instead, in this work we propose an automatic sizing procedure based on computer vision techniques, capable of accurately estimating a great number of samples in a sensor that combines acoustic and optical data. As shown in Figure 1, Figure 3 and Figure 6, the automatic sizing process can be considered to be divided into two important stages: (1) the segmentation and isolated identification of the fish with respect to the background in the image and with respect to the background and other traces in the acoustic echograms, and (2) the estimation for measurements of these fish combining 2D information to provide 3D information. The results show that 75 h of computing time are needed to analyze 83 h of recording, obtaining 5030 samples (60.6 samples per hour of computing). The proposed AO sensor and the procedure to automatically obtain fish measurements with lower computation time consumption, thanks to the processing of acoustic data instead of stereovision systems, could speed up the implementation of autonomous biomass estimation devices. Moreover, our system is able to extract a significant number of samples and to accurately size fish in two dimensions, SFL and W, which can be used to improve the biomass estimation.

We carried out our study in a controlled environment containing 77 ABFT juveniles in a sea water tank with lengths of between 0.50 and 1 m and in a range between 2 and 6 m, but further tests will be done in other scenarios such as biomass estimation in grow-out cages with adult ABFT and in transfers between cages. In further developments, the acoustic and optical devices will be embedded in the proposed AO sensor and the electronics and processing capabilities will be optimized to have an autonomous power supply and to enable real-time processing. For example, a buoy can be used to accommodate the electronics and the solar panels in a compact assembly, and the code implementation should be ported to C/C++ to improve performance. Other future developments are improving image segmentation procedures using pre-processing techniques, such as dehazing [48]; or deep learning techniques to remove unnecessary add-ons like back panels, working on other perspectives in addition to the ventral one, and more.

## Figures and Tables

**Table 1:** Identification of tilted (|θ| > 10°) and non-tilted (|θ| ≤ 10°) samples using Swimming Tilt Indicator (STI) and a Convolutional Neural Network (CNN) to dismiss tilted samples in the acoustic-optical (AO) measurements. NT = Number of tilted and non-tilted images used for network training.

		Tilted Samples |θ| > 10°		Non-Tilted Samples |θ| ≤ 10°
		True Positives	False Positives	True Positives
STI	STI∈[−0.4,0.4]	522/828 (63%)	306/828 (37%)	3486/5203 (67%)
	STI∈[−0.3,0.3]	588/828 (71%)	240/828 (29%)	2914/5203 (56%)
CNN	NT = 200	540/628 (86%)	88/628 (14%)	4253/5003 (85%)
	NT = 50	650/778 (83.5%)	128/778 (16.5%)	4225/5153 (82%)

**Table 2:** True positives of tilted (|θ| > 10°) and non-tilted (|θ| ≤ 10°) samples using Swimming Tilt Indicator (STI) and a Convolutional Neural Network (CNN) grouped by months. NT: Number of tilted and non-tilted images used for network training.

		MAY		SEPTEMBER	
		|θ| > 10°	|θ| ≤ 10°	|θ| > 10°	|θ| ≤ 10°
STI	STI∈[−0.4,0.4]	62.2%	67.2%	63.8%	66.7%
	STI∈[−0.3,0.3]	70.2%	56.7%	71.7%	55.4%
CNN	NT = 200	87.7%	86.9%	84.5%	82.3%
	NT = 50	82.4%	83.5%	84.6%	79.4%

**Table 3:** Comparison between acoustic–optical (AO) sensor and stereoscopic system in terms of number of measurements and computing time. NM: number of measurements; NMHR: number of measurements per hour of recording; NMHC: number of measurements per hour of computing.

	MAY		SEPTEMBER		TOTAL	
	AO SENSOR	STEREO SYSTEM	AO SENSOR	STEREO SYSTEM	AO SENSOR	STEREO SYSTEM
Recording time	33 h		50 h		83 h	
NM	2894	11,038	2136	10,603	5030	21,641
NMHR	88 samples/h	335 samples/h	42.7 samples/h	212 samples/h	60.6 samples/h	261 samples/h
Computing time	30 h	924 h (38.5 days)	45 h	1400 h (58.3 days)	75 h	2324 (96.8 days)
NMHC	96.5 samples/h	11.9 samples/h	47.5 samples/h	7.6 samples/h	67.1 samples/h	9.3 samples/h

**Table 4:** Statistical comparison between acoustic–optical (AO) sensor and stereoscopic system. NM: number of measurements; µ: population mean; σ: standard deviation; σ^2^: variance.

		MAY		SEPTEMBER	
		STEREO SYSTEM	AO SENSOR	STEREO SYSTEM	AO SENSOR
NM		11,038	2894	10,603	2136
SFL	µ	0.59	0.59	0.71	0.72
	σ	0.0761	0.0676	0.0907	0.0930
	σ^2^	0.0058	0.0046	0.0082	0.0086
W	µ	0.12	0.12	0.15	0.15
	σ	0.0170	0.0154	0.0228	0.0225
	σ^2^	2.88 × 10^−4^	2.4 × 10^−4^	5.20 × 10^−4^	5.06 × 10^−4^
